# To Medical Students

**Published:** 1888-07

**Authors:** 


**To Medical Students.**—The Southern Medical College, Atlanta, Georgia, is now regarded as among the very best Institutions in the United States. The College building is commodious and well arranged. The Chairs are well and ably filled. The curriculum is complete. The Dissecting-room is large and well-fitted up for the purpose. There is a Hospital connected with the College, and every facility exists for imparting a thorough medical education. The Annual Catalogue is now being distributed. Parties who desire a Catalogue should address, W. P. NICOLSON, M. D., DEAN, Atlanta, Ga.

SANDERS & SONS' EUCALYPTI EXTRACT (*Eucalyptol*).—Apply to Dr. Sanders, Dillon, Iowa, for gratis supplied reports on cures effected at the clinics of the Universities of Bonn and Griefswald.

